# Isolated displaced non-union of a triquetral body fracture: a case report

**DOI:** 10.1186/1752-1947-6-54

**Published:** 2012-02-10

**Authors:** Sonia Rasoli, Matthew Ricks, Greg Packer

**Affiliations:** 1 Trauma and Orthopaedic Department, Southend University Hospital, Westcliff on Sea, Southend, UK

## Abstract

**Introduction:**

Fractures of the body of the triquetral bone are the second most common carpal fractures, and these fractures can be missed on plain X-ray. Although non-union of triquetral body fractures is very rare, such cases are associated with considerable morbidity and reduction in functional activity.

**Case presentation:**

We report the case of a 29-year-old Caucasian British man who sustained an isolated displaced triquetral body fracture that resulted in non-union, who was treated surgically. We describe an original operative management for this debilitating injury. An open reduction and internal fixation using double headed compression screws was performed, without bone grafting, and with early immobilization of the wrist.

**Conclusions:**

We propose this novel approach and advocate early clinical suspicion of triquetral body fractures in patients with a history of fall on an outstretched hand and ulnar sided wrist pain. We recommend evaluation using computed tomography or magnetic resonance imaging scanning.

## Introduction

Triquetral fractures are the second most common carpal fractures after fractures of the scaphoid. There are two broad types, those involving the dorsal aspect of the bone (chip fractures), and those involving the body. Triquetral body fractures are less common of the two, and these fractures can be associated with non-union. Although non-union of triquetral body fractures is rare, with only three cases described so far in the medical literature [1-3], they are associated with considerable morbidity and reduction in functional activities. These fractures can also be missed on plain X-rays [4].

We report a case of displaced isolated triquetral body fracture that resulted in non-union, treated successfully with open reduction and internal fixation using compression screws without bone grafting, and with early mobilization of the wrist. To the best of our knowledge, this scenario has not been previously described in the literature.

## Case presentation

**Figure 1:**
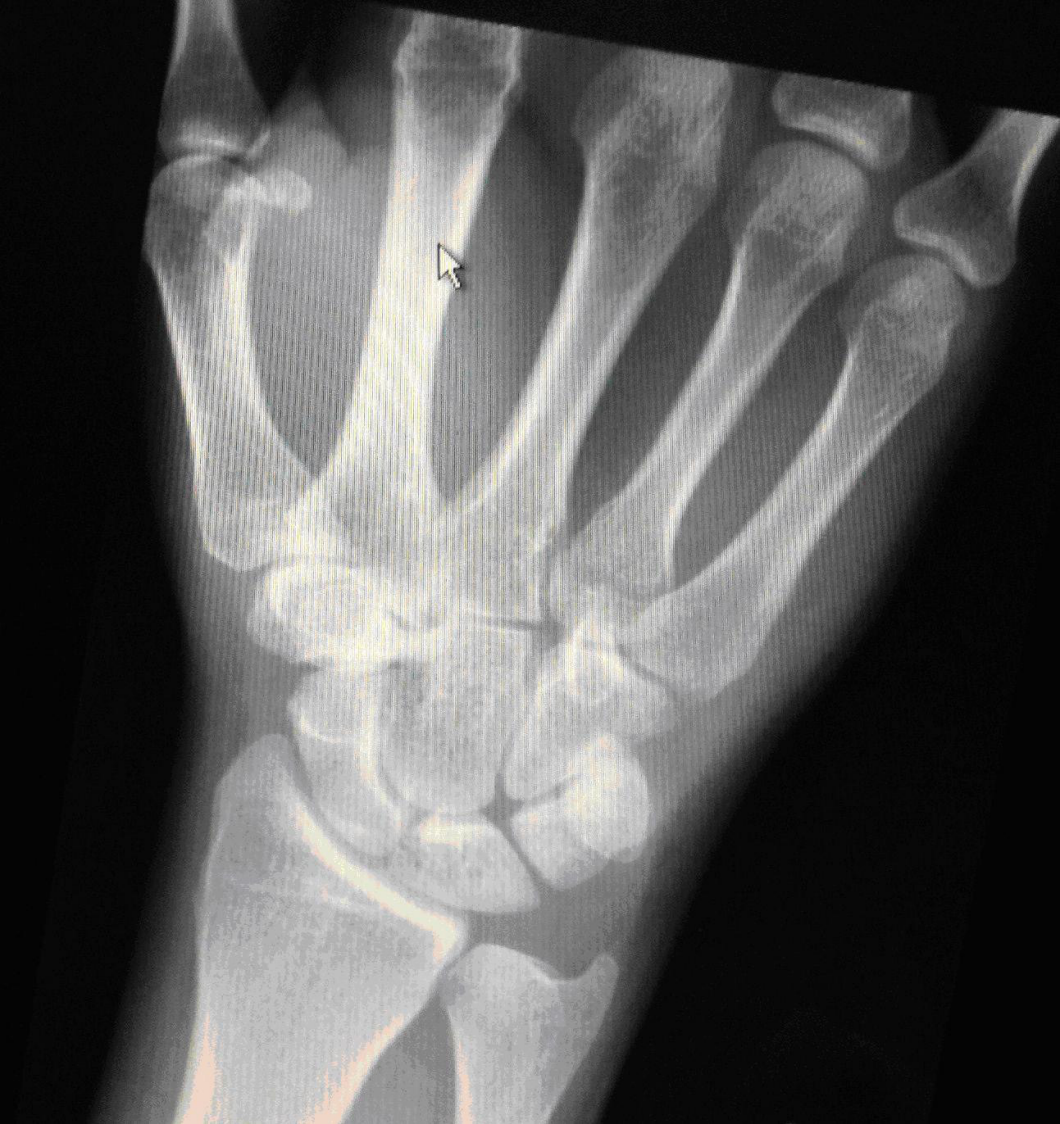
**Posteroanterior plain radiograph of the right wrist at six weeks after the injury**. The image shows no obvious evidence of bony injury.

**Figure 2:**
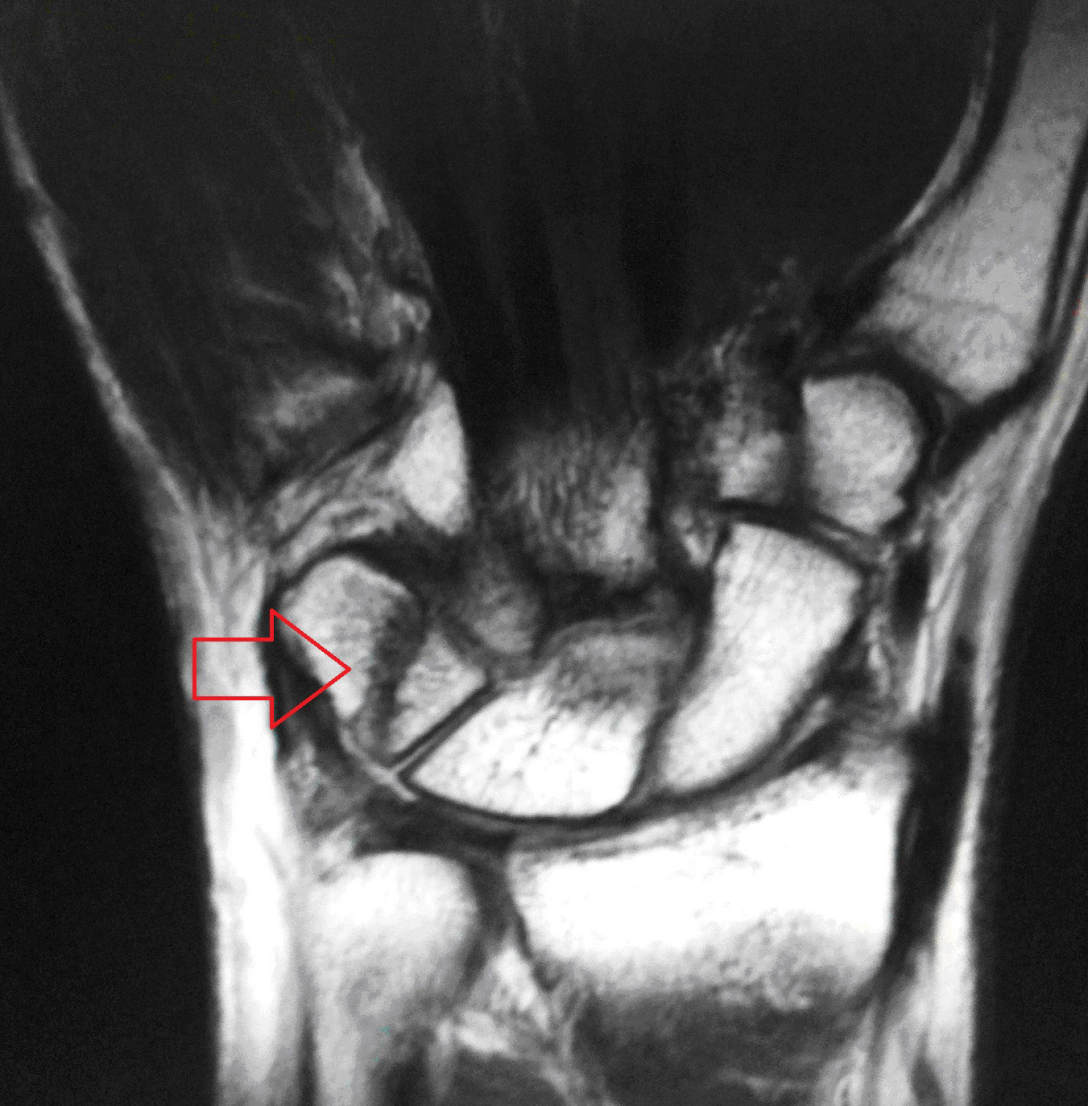
**Coronal magnetic resonance imaging scan of the right wrist at five months after the injury**. The image shows a non-united, displaced, and isolated fracture of the body of the triquetrum.

**Figure 3:**
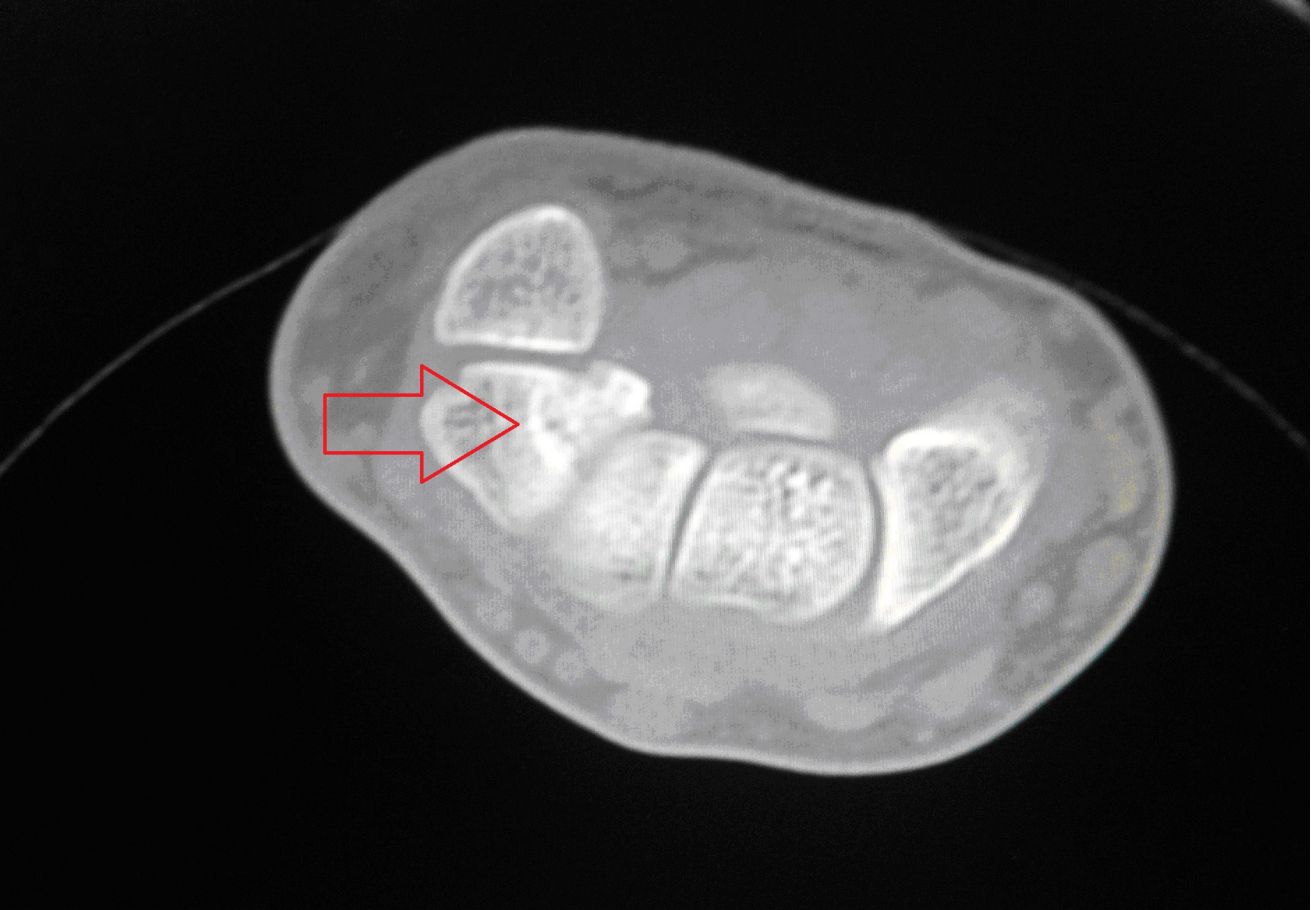
**Axial computerized tomography (CT) at six months after the injury**. The image shows a non-united, displaced, and isolated fracture of the body of the triquetrum.

**Figure 4:**
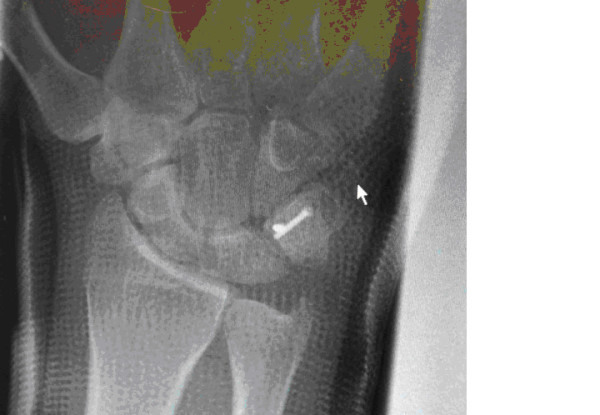
**Posteroanterior radiograph of the right wrist at six weeks post-operation**. The image shows no evidence of triquetral body non-union, and compression screws *in situ*.

A 29-year-old Caucasian British man fell onto his outstretched right hand with his wrist in dorsiflexion while playing football, and injured his wrist. He presented to our clinic three weeks after the fall complaining of persistent pain in his wrist. Examination of the wrist found discrete tenderness over the ulnar aspect of the wrist. There was no evidence of instability of the wrist, and a grind test was unremarkable for triangular fibrocartilage complex tear. No bony injuries were detected on plain X-ray (Figure 1). A diagnosis of soft tissue injury to the wrist was suspected and our patient treated with wrist immobilization with a Futuro^® ^wrist splint. Our patient continued to remain symptomatic with tenderness over the ulnar aspect of his wrist for four months after the injury, despite the treatment. At this point an a magnetic resonance imaging (MRI) scan was carried out, which suggested a non-union and minimally displaced fracture of the triquetral body (Figure 2). No other deformities were found. A computed tomography (CT) scan confirmed the diagnosis (Figure 3). Our patient finally underwent an open reduction and surgical fixation at six months after the injury due to persistence of symptoms. A dorsal approach was used to access the triquetrum with a 3 cm incision made between the third and fourth metacarpal. Dissection was carried out to expose the triquetrum and the non-union fragments were visualized. The fragments were reduced under direct vision and fixed with two headless, mini-compression screws (Stryker Ltd). No bone graft was used and the wrist was mobilized immediately after surgery. Our patient was followed-up at two weeks, six weeks, and 12 weeks post-operatively to union. The fracture went on to complete union radiographically (Figure 4). Our patient returned to work, with resumption of his pre-injury activity level.

## Discussion

Although non-union of triquetral body fractures is rare, such cases can lead to considerable disability. After extensive systematic review of the literature, searching Embase, Medline, Cochrane, Cinhal, and Google search engines, we could identify only three previous reports of non-union of triquetral body fracture [1-3]. This low incidence could be attributed to the rich vascular supply of the triquetrum, which may explain the low risk of developing avascular necrosis in these fractures. Durbin [2] reported treating triquetral non-union with immobilization in plaster cast, which was unsuccessful and the patient remained symptomatic. Abboud *et al. *initially treated the non-union with cast immobilization, which did not respond, and subsequently carried out an open reduction and internal fixation using headless compression screws, with iliac bone autograft [1]. Kawakami *et al. *also achieved successful bone reunion of the triquetral fragments with open reduction and internal fixation using headless compression screws and iliac bone graft [3].

In our patient, we carried out open reduction and internal fixation, but we did not use a bone graft. Kawakami *et al. *further treated their patient with eight weeks post-surgical immobilization [3]. In our patient, we did not use post-surgical immobilization. In our patient the fracture was missed on radiograph and a diagnosis made by CT scan (Figure 3). A previous report found that only 20% of triquetral fractures were shown on radiographs [4].

## Conclusions

Given the morbidity associated with non-union of triquetral body fractures we encourage a high index of suspicion for these fractures in people who have fallen on an outstretched hand with ulnar sided wrist pain. We recommend that patients with persistent ulnar sided pain and disability should be further investigated for this injury. CT and MRI have a high sensitivity in allowing visualization of both bony and soft tissue injury and morphology of the fracture to determine the need for early fixation and to reduce morbidity in this group of patients.

## Consent

Written informed consent was obtained from the patient for publication of this case report and any accompanying images. A copy of the written consent is available for review by the Editor-in-Chief of this journal

## Competing interests

The authors declare that they have no competing interests.

## Authors' contributions

SR wrote the majority of the case report with MR writing a substantial part and being responsible for referencing and editing. GP was the lead clinician in charge of our patient, and performed the operation and follow-up. GP was also involved in the conception and editing of the case report. All authors read and approved the final manuscript.

## References

[B1] Abboud JA Beredjikilian PK Bozenka DJ Nonunion of a triquetral body fracture. A case report J Bone Joint Surg Am 2003 85-A 2441 2444 14668518 10.2106/00004623-200312000-00026

[B2] Durbin FC Non-union of the triquetrum J Bone Joint Surg Br 1950 32 388 14778860 10.1302/0301-620X.32B3.388

[B3] Kawakami Y Fujioka H Kurosaka M Treatment of non-union of a triquetral body fracture J Hand Surg Br 2007 32 717 718 10.1016/J.JHSE.2007.05.011 17993442

[B4] Welling RD Jacobson JA Jamadar DA Ching S Caoili EM Jebson PJ MDCT and radiography of wrist fractures: radipgraphic sensitivity and fracture patterns AJR Am J Roentgenol 2008 190 10 16 10.2214/AJR.07.2699 18094287

